# Association between lipids profile and thyroid parameters in euthyroid diabetic subjects: a cross-sectional study

**DOI:** 10.1186/s12902-015-0008-3

**Published:** 2015-03-27

**Authors:** Yun Zhang, Ping Lu, Ling Zhang, Xinhua Xiao

**Affiliations:** Department of Endocrinology, Henan Province People’s Hospital & Zhengzhou University People’ Hospital, Zhengzhou City, Henan Province P R China; Department of Endocrinology, Peking Union Medical College Hospital, Chinese Academy of Medical Sciences & Peking Union Medical College, 100730 Beijing, P R China

**Keywords:** Total cholesterol level, Thyroid-stimulating hormone, Euthyroidism, Diabetes, Thyroid antibodies

## Abstract

**Background:**

The concept is now emerging that higher thyroid-stimulating hormone (TSH) and lower thyroid hormone levels within the euthyroid range may adversely affect atherosclerosis. The aim of this study was to investigate the potential association between thyroid parameters and lipids profile in a cohort of euthyroid diabetic subjects.

**Methods:**

Four hundred and sixty-two euthyroid type 2 diabetes subjects (302 males and 160 females) were consecutively recruited. Clinical and anthropometric data was collected from all participants. Whole blood samples were drawn in the morning after an overnight fasting for the measurement of serum TSH, free thyroxine (FT4), free triiothyronine (FT3), anti-thyroid peroxidase antibody (TPO-Ab) levels, as well as lipid concentrations and glucose.

**Results:**

TSH was higher in females than males. Stratified by TSH, high-density lipoprotein cholesterol (HDL-c) level increased in subjects with TSH ≥2.5uIU/mL (P = 0.004). And TSH was associated with HDL-c in a Pearson correlation test, however, the association failed to attain significance in partial correlation analyses, adjusted for age, sex, duration of diabetes, fasting glucose and BMI. In females, total cholesterol (TC) and low-density lipoprotein cholesterol (LDL-c) level was significant lower in subjects with TSH <2.5uIU/mL. TSH was significantly associated with TC and LDL-c, even in a partial correlation analysis (P = 0.006 and 0.011, respectively). In a multiple linear regression analysis (stepwise), TSH was positive associated with TC (β = 0.202, P = 0.005) and LDL-c (β = 0.144, P = 0.010). In one hundred and six patients having TPO antibody assays, 6 (5.66%) were positive. The blood pressure and lipid levels were lower in TPO-Ab positive patients, however, the differences were not significantly.

**Conclusions:**

In conclusion, we identified TSH was positively associated with serum TC and LDL-c in euthyroid diabetic women. Our analysis in the subgroup having TPO antibody assays demonstrating non-significantly lower TC levels among seropositive subjects was consistent with the above stated consideration for women as a whole. Further investigations are needed to understand the intimate mechanisms of lipid metabolism in type 2 diabetes with respect to thyroid function.

## Background

Thyroid hormones were recognized as catabolic hormones and they regulated various processes of metabolism, including the synthesis, mobilization, and breakdown of lipids. Hypothyroidism had been reported to be associated with an increased risk for dyslipidemia and atherosclerotic cardiovascular disease [1,2]. Interestingly, the concept was emerging nowadays that effects of low thyroid function on atherosclerosis susceptibility might extend into the euthyroid range. Several studies reported an association between higher thyroid-stimulating hormone (TSH) and lower thyroid hormone levels that were still within the normal range and lipids profile in the euthyroid population [3,4].

Diabetes mellitus, in particular type 2 diabetes, which was mostly associated with lipoid abnormalities [5], was also known to dramatically increased risk of cardiovascular diseases [6]. Notably, thyroid dysfunctions were more frequent in diabetic patients than in the general population [7]. Moreover, the association between circulating TSH levels and cardiovascular diseases risk factors seemed to be amplified by the degree of insulin-resistance [8], and it might be particularly relevant in type 2 diabetes. Thus, glucose, lipids, and thyroid hormones seemed to interact according to a more complex mathematical function than as previously expected. Recently, several studies had reported that even relative low thyroid functions that were still within normal range were more frequent and might be more dangerous in people with diabetes [9-11]. However, only scanty studies on these matters were available. And in most of these studies, free triiodothyronine (FT3), free thyroxine (FT4) and TSH were not measured together.

Taking into account the consideration mentioned above, with the present study, we investigated the potential association between TSH and thyroid hormones levels within the normal range and lipids profile in a cohort of euthyroid type 2 diabetes subjects. We hoped that the information from this study would lead to a better understanding of the relationship between thyroid parameters and lipids profile.

## Methods

### Subjects

Four hundred and sixty-two euthyroid type 2 diabetes subjects (302 males and 160 females) were consecutively recruited in this cross-sectional study from inpatients of Department of Endocrinology, Henan Province People’s Hospital. Type 2 diabetes was diagnosed according to American Diabetes Association 2009 criteria [12]. Euthyroidism was defined as TSH, FT3, and FT4 levels within their normal reference ranges (see the next paragraph). Exclusion criteria: type 1 diabetes, latent immune diabetes of the adults, gestational diabetes, and other type of diabetes, pregnancy, neoplasms, as well as any major medical condition in the 6 months preceding the study (i.e. liver, kidney, and heart failure). In particular, subjects with a previous history of thyroid diseases, such as overt hyper/hypothyroidism, thyroid cancer, were excluded. Subjects taking medications affecting thyroid hormone levels (such as thyroid supplementation and antithyroid agents, IFNc, amiodarone, lithium, corticosteroids, etc.) and lipids profile (such as statins, fenofibrate, etc.) were also excluded. Informed consent was obtained from all participants. The present study was approved with the Institutional Ethnics Committee in Zhengzhou University.

### Clinical, anthropometric, and laboratory measurements

Clinical data was collected from all participants. Weight and height were measured with the subjects wearing light clothing and no shoes; the body mass index (BMI) was also calculated (kg/m^2^). Whole blood samples were drawn in the morning after an overnight fasting for measurements of the study parameters. Serum TSH, FT3, and FT4 levels were measured using chemiluminescence tests (SIEMENS Advia Centaur XP). The normal ranges were as follows: FT3 3.5-6.5pmol/L; FT4 11.5-22.7pmol/L; TSH 0.55-4.78uIU/mL. Anti-thyroid peroxidase antibody (TPO-Ab) was measured in 106 patients with agreement, with reference values between 0 and 60U/L. The normal range of all these thyroid parameters came from 1,000 normal controls determined before this study. And the inter-assay and intra-assay imprecisions (CV) were all less than 5%. Fasting plasma glucose (FPG) and lipid concentrations (total cholesterol (TC), triglycerides (TG), high-density lipoprotein cholesterol (HDL-c), and low-density lipoprotein cholesterol (LDL-c)) were assayed using enzymatic methods (Roche Diagnostics).

### Statistical analyses

The data analysis was performed using SPSS version18.0. All data was expressed as means ± standard deviation (SD). All subjects were divided into two groups by sex or TSH (≥2.5uIU/mL and <2.5uIU/mL). Student’s *t* test or Mann–Whitney U test, depending on the shape of the distribution curves, was used for evaluation of differences between the two groups. The Pearson correlation test was applied in order to assess for the existence of any significant interdependence between numerical parameters. Partial correlation analyses were performed to evaluate the association of thyroid parameters with major cardiovascular risk factors (blood pressure and lipid concentrations), adjusted for age, sex, duration of diabetes, FPG, and BMI. Furthermore, we performed a multiple linear regression analysis (stepwise) to confirm the association, with cardiovascular risk factors as dependent variables, independent variables as follows: age, sex, BMI, duration of diabetes, FPG, FT3, FT4, TSH. Males and females were analyzed separately. Subjects with data of TPO-Ab were divided into two groups: TPO-Ab positive (TPO-Ab >60U/L) and TPO-Ab negative (TPO-Ab <60U/L), and differences between the two groups were compared by *t* tests.

## Results

Table 1 showed the clinical characteristics of this sample of 462 diabetic patients with euthroidsm. The mean age was 54.36 ± 11.85 years (range 27 to 84), and the mean BMI was 25.33 ± 3.47 kg/m^2^ (range 17.5 to 37.2). Age and TSH level were higher in females, and BMI, diastolic blood pressure (DBP), and FT3 were lower. No difference was found in FT4 or duration of diabetes.

**Table 1:** **Clinical characteristics of the subjects**

	**Total**		**Males**		**Females**	
n	462		302		160	
Age(years)	54.36 ± 11.85		53.20 ± 11.87		56.56 ± 11.52	
Duration of diabetes(years)	8.33 ± 6.97		8.36 ± 7.10		8.27 ± 6.74	
BMI (kg/m^2^)	25.33 ± 3.47		25.61 ± 3.10		24.80 ± 4.03	
FT3(pmol/L)	4.49 ± 0.49		4.62 ± 0.49		4.24 ± 0.38	
FT4(pmol/L)	16.60 ± 2.29		16.74 ± 2.27		16.34 ± 2.32	
TSH(uIU/mL)	2.05 ± 0.95		1.97 ± 0.89		2.19 ± 1.03	
	TSH ≥ 2.5 (n = 120)	TSH < 2.5 (n = 342)	TSH ≥ 2.5 (n = 69)	TSH < 2.5 (n = 233)	TSH ≥ 2.5 (n = 51)	TSH < 2.5 (n = 109)
SBP(mmHg)	133.10 ± 14.54	133.52 ± 17.52	132.86 ± 14.22	133.56 ± 18.04	133.43 ± 15.11	133.42 ± 16.45
DBP(mmHg)	82.51 ± 8.89	82.41 ± 9.99	84.14 ± 8.26	83.42 ± 10.27	80.29 ± 9.32	80.24 ± 9.03
TC (mmol/L)	4.88 ± 1.12	4.82 ± 1.06	4.67 ± 1.15	4.86 ± 1.12	5.16 ± 1.02	4.74 ± 0.92*
TG (mmol/L)	2.04 ± 2.47	2.11 ± 1.81	2.35 ± 3.13	2.29 ± 2.01	1.62 ± 0.94	1.72 ± 1.17
HDL-c (mmol/L)	1.18 ± 0.30	1.09 ± 0.27*	1.11 ± 0.28	1.07 ± 0.27	1.27 ± 0.31	1.14 ± 0.26
LDL-c (mmol/L)	2.88 ± 0.84	2.90 ± 0.83	2.76 ± 0.85	2.95 ± 0.88	3.04 ± 0.81	2.81 ± 0.71*
FBG (mmol/L)	8.55 ± 3.50	8.97 ± 3.74	8.51 ± 3.34	8.96 ± 3.84	8.60 ± 3.74	9.00 ± 3.52

*P < 0.05, when compared with TSH ≥ 2.5umIU/mL.

Stratified by TSH, HDL-c level increased in subjects with TSH ≥2.5uIU/mL (P = 0.004, Table 1). And TSH was associated with HDL-c in a Pearson correlation test (β = 0.095, P = 0.042), however, the association failed to attain significance in partial correlation analyses (P = 0.092, Table 2), adjusted for age, sex, duration of diabetes, fasting glucose and BMI.

**Table 2:** **Correlation of thyroid parameters with lipids and blood pressure in Partial correlation analyses**

		**FT3**		**FT4**		**TSH**	
		**coefficients**	**P**	**coefficients**	**P**	**coefficients**	**P**
Total	SBP	0.027	0.566	−0.006	0.907	−0.026	0.588
	DBP	0.035	0.453	−0.009	0.845	0.017	0.719
	TC	0.012	0.794	0.010	0.835	0.044	0.352
	TG	0.034	0.472	0.004	0.933	−0.003	0.948
	HDL-c	0.044	0.347	0.033	0.484	0.079	0.092
	LDL-c	0.001	0.978	−0.018	0.699	0.020	0.668
Males	SBP	0.028	0.630	−0.043	0.466	−0.046	0.430
	DBP	0.015	0.796	−0.032	0.582	0.000	0.999
	TC	−0.004	0.945	0.041	0.487	−0.059	0.317
	TG	0.010	0.867	0.011	0.847	−0.043	0.467
	HDL-c	0.007	0.900	0.083	0.154	0.062	0.288
	LDL-c	−0.008	0.885	−0.017	0.773	−0.080	0.171
Females	SBP	0.022	0.787	0.064	0.433	0.001	0.992
	DBP	0.103	0.206	−0.017	0.835	0.047	0.561
	TC	−0.010	0.905	−0.043	0.596	0.221	0.006*
	TG	0.022	0.786	−0.051	0.530	0.050	0.537
	HDL-c	0.147	0.069	−0.064	0.431	0.113	0.163
	LDL-c	−0.038	0.642	0.002	0.982	0.204	0.011*

*P < 0.05.

In females, TC and LDL-c level was significant lower in subjects with TSH <2.5uIU/mL (Table 1). TSH was significantly associated with TC and LDL-c, even in a partial correlation analysis (P = 0.006 and 0.011, respectively, Table 2). In a multiple linear regression analysis (stepwise), TSH was positive associated with TC (β = 0.202, P = 0.005) and LDL-c (β = 0.144, P = 0.010). In males, no difference was found in lipids profile or BP between subjects with TSH ≥2.5uIU/mL and TSH <2.5uIU/mL group (Table 1).

In one hundred and six patients with TPO-Ab measured, 6 (5.66%) were positive and 100 (94.34%) were negative. In TPO-Ab positive patients, SBP, DBP, FT3, TC, TG, HDL, LDL levels were lower than TPO-Ab positive patients. However, the differences were not significantly (Table 3).

**Table 3:** **Clinical characteristics of the subjects with data of TPO-Ab**

	**TPO-Ab negative**	**TPO-Ab positive**	**P**
N(males/females)	100 (71/29)	6 (3/3)	
Age(years)	52.71 ± 11.76	59.67 ± 10.03	0.160
BMI (kg/m^2^)	25.76 ± 3.38	23.17 ± 2.21	0.160
SBP (mmHg)	133.84 ± 16.99	126.00 ± 13.55	0.271
DBP (mmHg)	82.50 ± 9.68	77.83 ± 8.30	0.251
FT3(pmol/L)	4.54 ± 0.45	4.37 ± 0.45	0.353
FT4(pmol/L)	16.30 ± 2.16	16.69 ± 2.16	0.682
TSH(uIU/mL)	2.21 ± 1.13	2.49 ± 1.15	0.588
TC (mmol/L)	4.95 ± 1.15	4.30 ± 0.48	0.176
TG (mmol/L)	2.06 ± 2.05	1.59 ± 1.31	0.578
HDL-c (mmol/L)	1.17 ± 0.32	0.95 ± 0.27	0.099
LDL-c (mmol/L)	3.05 ± 0.94	2.75 ± 0.40	0.444
FBG (mmol/L)	8.40 ± 3.26	7.74 ± 5.48	0.647

## Discussion

Thyroid dysfunction was a risk factor for cardiovascular disease mediated by the effects of thyroid hormones on lipids metabolism and blood pressure [13-15], yet most subjects at risk for cardiovascular disease were euthyroid in the clinical setting. The relationship between thyroid hormones and atherosclerosis in the euthyroid population had garnered much interest recently.

In this study, we demonstrated that TSH was higher in females than males, which was in agreement with previous studies [9]. Furthermore, we found that in diabetic females, TC and LDL-c level was significant lower in subjects with TSH <2.5uIU/mL. TSH was significantly associated with TC and LDL-c, even in a partial correlation analysis. In a multiple linear regression analysis (stepwise), TSH was positive associated with TC and LDL-c. TSH levels within the reference range had been reported to be associated with serum lipid concentrations previously. Giandalia et al. reported that TSH was associated with visceral obesity and higher triglycerides concentration in type 2 diabetes [9]. And higher TSH was reported to confer increased plasma cholesteryl ester transfer in the context of chronic hyperglycemia in Triolo’s study [11]. All these suggested that relatively low but clinically normal thyroid function, as inferred from higher TSH in normal range, could also influence lipids profile and atherosclerosis susceptibility in type 2 diabetes.

One explanation to the positive association of TSH with TC and LDL-c might be both being a consequence of autoimmune activation involving lipoprotein(a), with ensuing “reduced” lipoprotein(a) levels, a determinant of new-onset diabetes, and accompanied by low circulating TC and LDL-c and autoimmune complex involving TSH as well. Among euthyroid patients with established diabetes, the stated variables might tend to normalize secondary to a decline in autoimmune processes and in the reduction of lipoprotein(a) levels [16]. And lower TSH in diabetic women associated with lower cholesterol could be attributed to two phenomena: a) Lower TC and LDL-c commonly precede autoimmune-initiated processes –be it oxidatively-damaged TSH or rheumatoid arthritis [17] or diabetes [18]; b) TSH incurring epitope damage escapes immunoassay and is measured as “lower”.

Some authors suggested that chronic autoimmune thyroiditis per se might be considered as a risk factor of atherosclerosis independent of thyroid function [19]. In this study, TPO-Ab was considered for the first time in the association of thyroid function and lipids profile and blood pressure in diabetic subjects. Along with higher TSH and lower FT3 values in the seropositive group, lower lipid concentrations and blood pressure were observed, although the differences were not significantly. The result did not support autoimmune thyroiditis as a risk factor for atherosclerosis. This might result from the small sample size, only 6 patients with TPO-Ab positive. Larger scale studies were needed to further confirm the role of thyroid antibodies in atherosclerosis.

Several methodological aspects and limitations of our study needed to be considered. First of all, the causal relationship could not be inferred from this study because it was cross-sectional in nature. Secondly, in type 2 diabetes patients, the relationship between thyroid hormones and cardiovascular disease risk might be influenced by other diabetes-related variables, such as metabolic control, co-morbidities, and/or hypoglycemic therapies. Strollo et al. found that TC and LDL-C correlated negatively with FT4 and positively with FT3 only in patients treated with insulin, but not in patients treated with oral hypoglycaemic agents [19]. However, treatment of diabetes was not considered in this study.

## Conclusion

In conclusion, we identified TSH was positively associated with serum TC and LDL-c in euthyroid diabetic women. Our analysis in the subgroup having TPO antibody assays demonstrating non-significantly lower TC levels among seropositive subjects was consistent with the above stated consideration for women as a whole. Further investigations are needed to understand the intimate mechanisms of lipid metabolism in type 2 diabetes with respect to thyroid function.

## Abbreviations

TSH: Thyroid-stimulating hormone
FT3: Free triiodothyronine
FT4: Free thyroxine
BMI: Body mass index
TPO-Ab: Anti-thyroid peroxidase antibody
TC: Total cholesterol
TG: Triglycerides
HDL-c: High-density lipoprotein cholesterol
LDL-c: Low-density lipoprotein cholesterol
SD: Standard deviation
DBP: Diastolic blood pressure
SBP: Systolic blood pressure
